# Exploring RNA cargo in extracellular vesicles for pleural mesothelioma detection

**DOI:** 10.1186/s12885-025-13617-y

**Published:** 2025-02-07

**Authors:** Agnieszka Kraft, Michaela B. Kirschner, Vanessa Orlowski, Manuel Ronner, Caroline Bodmer, Valentina Boeva, Isabelle Opitz, Mayura Meerang

**Affiliations:** 1 Department of Thoracic Surgery, University Hospital Zurich, Raemistrasse 100, 8091 Zurich, Switzerland; 2 Institute for Machine Learning, Department of Computer Science, ETH Zurich, Zurich, Switzerland; 3 Swiss Institute of Bioinformatics (SIB), Zurich, Switzerland; 4 University of Zurich, Zurich, Switzerland; 5 ETH AI Center, ETH Zurich, Zurich, Switzerland; 6 UMR 8104, UMR-S1016, Cochin InstituteCNRSParis Descartes University, Inserm U1016, 75014 Paris, France

**Keywords:** Pleural mesothelioma, Biomarkers, Extracellular vesicles, Transcriptomics, Diagnosis

## Abstract

**Background:**

Pleural Mesothelioma (PM) is a highly aggressive cancer, for which effective early detection remains a challenge due to limited screening options and low sensitivity of biomarkers discovered so far. While extracellular vesicles (EVs) have emerged as promising candidates for blood-based biomarkers, their role in PM has not been studied yet. In this study, we characterized the transcriptomic profile of EVs secreted by PM primary cells and explored their potential as a biomarker source for PM detection.

**Methods:**

We collected cell culture supernatant from early-passage PM cell cultures derived from the pleural effusion of 4 PM patients. EVs were isolated from the supernatant using Qiagen exoEasy Maxi kit. RNA isolation from EVs was done using the mirVana PARIS kit. Finally, single-end RNA sequencing was done with Illumina Novaseq 6000.

**Results:**

We identified a range of RNA species expressed in EVs secreted by PM cells, including protein-coding RNA (80%), long non-coding RNA (13%), pseudogenes (4.5%), and short non-coding RNA (1.6%). We detected a subset of genes associated with the previously identified epithelioid (32 genes) and sarcomatoid molecular components (36 genes) in PM-EVs. To investigate whether these markers could serve as biomarkers for PM detection in blood, we compared the RNA content of PM-EVs with the cargo of EVs isolated from the plasma of healthy donors (publicly available data). Majority of upregulated genes in PM-EVs were protein-coding and long non-coding RNAs. Interestingly, 25 of them were the sarcomatoid and epithelioid marker genes. Finally, functional analysis revealed that the PM-EV RNA cargo was associated with Epithelial-Mesenchymal transition, glycolysis, and hypoxia.

**Conclusions:**

This is the first study to characterize the transcriptomic profile of EVs secreted by PM primary cell cultures, demonstrating their potential as biomarker source for early detection. Further investigation of the functional role of PM-EVs will provide new insights into disease biology and therapeutic avenues.

**Supplementary Information:**

The online version contains supplementary material available at 10.1186/s12885-025-13617-y.

## Background

Pleural Mesothelioma (PM) is an aggressive cancer of the mesothelium covering the internal lining of the lungs, known as pleura. It is primarily caused by asbestos exposure, and despite stringent regulations on asbestos use in most countries worldwide, the incidence of PM is expected to peak in the coming decade [1, 2]. Treatment of PM remains an imposing challenge, with most patients facing a bleak prognosis of only 9–19 months post-diagnosis [3–5]. One of the key factors affecting the prognosis of PM patients is the cell type composition of the tumor. The two histological subtypes, epithelioid and sarcomatoid, characterized by the prevalence of epithelioid (E) or sarcomatoid (S) cells, present varied responses to treatment. While more common epithelioid tumors respond better to treatment, sarcomatoid ones are less common and more aggressive. Additionally, some patients present a biphasic subtype, characterized by the coexistence of epithelioid and sarcomatoid cell types [3, 6, 7]. Recent large-scale studies on PM molecular profiling have revealed significant heterogeneity of the disease, represented by a continuum of molecular phenotypes ranging from *E* to *S*, characterized by scores of *E-* and *S-*gene signatures [8]. Interestingly, the spectrum of these molecular phenotypes has been observed even within morphologically defined epithelial cells [8–10]. These phenotypes are strongly associated with patient prognosis, as well as linked with specific DNA methylation patterns and the composition of tumor microenvironment [8, 9, 11, 12].

PM poses significant treatment challenges, with overall survival rates remaining low [3–5, 13], partly because PM patients are usually diagnosed at advanced stages and have limited treatment options. While recent advancements in immunotherapy, including nivolumab and ipilimumab, offer a promising avenue for enhancing patient survival, their efficacy has thus far been demonstrated primarily in non-epithelial tumors [6, 14, 15]. Therefore, the quest for new effective treatment targets and biomarkers for early disease detection remains essential for improving disease outcomes.

Numerous studies have focused on identifying PM biomarkers for routine screening of asbestos-exposed individuals for early disease detection. Considering the safety and non-invasive nature of blood tests, an ideal approach for early detection of PM would involve a blood-based biomarker, such as DNA, protein, and RNA, secreted by tumors. While various serum-based protein markers have been proposed for PM detection, their specificity and sensitivity for widespread screening remain unproven [16–27]. Therefore, the need for effective non-invasive biomarkers to detect PM remains unmet.

Extracellular vesicles (EVs) have been previously shown as a promising source of biomarkers. EVs, represented primarily by microvesicles, exosomes and apoptotic bodies [28, 29], carry a diverse cargo, including microRNA, mRNA, and DNA, which can be transferred between cells to facilitate intercellular communication and molecular exchange [30, 31]. Recent studies have highlighted the crucial role of EVs in mediating communication between cancer cells and their microenvironment, fostering tumor progression [32–36]. Consequently, EVs have also emerged as potential targets for treatment interventions. Moreover, EVs have arisen as a more sensitive method for detecting cancer-related biomarkers in blood compared to traditional liquid biopsy testing [37], positioning them as a promising strategy for noninvasive early cancer detection across multiple cancer types [38]. However, despite their significance, the role of EVs secreted by PM cells remains poorly understood.

Here, we characterized the transcriptomic profile of extracellular vesicles secreted by pleural mesothelioma primary cell cultures (PM-EVs). We analyzed the biotypes of genes expressed in PM-EVs and identified gene markers of PM intratumor heterogeneity secreted into the EV cargo. Our analysis revealed RNAs upregulated in PM-EVs compared to vesicles circulating in the plasma of healthy donors and linked their expression with increased activity of Epithelial-Mesenchymal transition, glycolysis, and hypoxia. Our work underlines the role of EV RNA cargo in PM biology and highlights its potential as a novel source of biomarkers and treatment targets for this disease.

## Materials and methods

### Cell culture

Primary cell cultures of four pleural mesothelioma cell cultures and three non-malignant mesothelial cell cultures were established using pleural effusion samples of patients undergoing diagnosis at the Department of Thoracic Surgery at the University Hospital Zurich (Table 1, Table S10). Pleural effusion was collected and cells were sedimented by centrifugation at 400 g for 5 min. After the removal of the supernatant, cells were incubated with a red blood cell lysis buffer for 5 min. After washing with DPBS, cells were cultivated in a gelatin coated flask with a RPMI medium ATCC modification (GibcoTM, A1049101, Life Technology Europe BV, Zug, Switzerland) containing 2 mM glutamine (Sigma-Aldrich Buchs, Switzerland) and 10% fetal bovine serum (FBS) (Biowest, Nuaillé, France), penicillin/streptomycin (Biowest), heparin 2 µg/ml (SigmaAldrich), hydrocortisone 2 µg/ml (SigmaAldrich), human Epidermal Growth Factor (20 ng/ml, hEGF) (Peprotech) and maintained at 37 °C and 5% CO2. All cell cultures were regularly tested for mycoplasma contamination.

**Table 1:** Clinical characteristics associated with the analyzed PM samples

**Sample code**	**Gender**	**Age at diagnosis**	**Histological subtype**	**Asbestos exposure**	**cT stage**^a^	**cN stage**^b^	**cN stage**^c^	**cM stage**^a^
PM-B1	male	58	biphasic	no	1	2	1	0
PM-B2	male	65	biphasic	yes	3	2	1	0
PM-B3	male	56	biphasic	yes	3	3	2	1
PM-E1	male	68	epithelioid	yes	3	3	2	1

^**+**^7th & 8th edition AJCC Cancer Staging Manual

^b^7th edition AJCC Cancer Staging Manual

^**c**^8th edition AJCC Cancer Staging Manual

### Immunohistochemistry

Tissue sections (2 μm) were deparaffinized and rehydrated. The staining for pan-Cytokeratin (AE1/AE3, Dako), Podoplanin (D2-40, Dako), Calretinin (DAK-Calret 1, Dako), WT1 (6F-H2, Dako) and BAP1 (C-4, Santa Cruz) was performed with Dako Autostainer Link48 Instrument (Dako Denmark A/S) according to the manufacturer's instructions.

### Copy number arrays

We isolated DNA from primary cell cultures using Qiagen DNeasy Blood & Tissue mini Kits (69504, Qiagen Hombrechtikon Switzerland). DNA from tumor tissues were isolated by Qiagen FFPE DNA isolation kit (56544, Qiagen). PM primary cells and matched tissue samples were characterized using Affymetrix OncoScan Microarray according to the manuals from the manufacturer by IMGM Laboratories GmbH (82152 Martinsried, Germany). Obtained CEL files were analyzed using Analysis Power Tools (Thermo Fisher, version 2.4.0) and rCGH R package. Our analysis confirmed a consistency between DNA copy number profiles of cultivated PM cells and matching tumors.

### EV isolation

Cells were grown in the complete medium in a 150 cm^2^ non-coated cell culture flask until reaching 80–90% confluence. Cells were then washed three times with 15 ml DPBS to clean EV contamination in the FBS. After that, 18 ml of the culture media without FBS was added. After 24 h, we collected the 18 ml supernatant and centrifuged it at 400 g for 5 min at 4ºC to sediment cells. The supernatant was subjected to centrifugation again at 2000 g for 15 min at 4ºC to remove large debris. Afterwards, the supernatant was filtered through 0.8 µm syringe driven filter (Corning 28 mm Syringe Filter, 0.8 μm, non-pyrogenic, sterile (Nr.:431221)) followed by EV isolation with ExoEasy Maxi kit according to the manufacturer’s instructions (76064, Qiagen). One milliliter of plasma samples were used for EV isolation. We centrifuged the plasma at 5,000 g for 20 min at 20 °C to pellet platelets. Platelet poor plasma was collected and centrifuged again at 10,000 g for 10 min at 20 °C to remove debris, followed by filtration with 0.8 µm syringe driven filter and EV isolation.

### Characterisation of isolated EVs

To understand the characteristics of EVs and the specificity of the kit, we performed quality control steps according to the guidelines [39]. Nanoparticle Tracking Analysis was performed using NanoSight (NS300-Malvern). Transmission electron microscopy was performed after negative staining of 20 µl of elute from the ExoEasy column, dried on glow-discharged grid 300 mesh copper, with 1% uranyl acetate for 1 min. For western blot, 18 ml cell culture supernatant was harvested. 14 ml fraction was subjected to EV isolation, all the EV eluate (500 µl) was further concentrated with Amicon Ultra 4 ml 3 K (UFC800324, Merck, Schaffhausen Switzerland) by centrifugation at 4000 g. 4 ml of supernatant was subjected to protein concentration using Amicon Ultra 4 ml 3 K. After centrifugation, the remaining volume of around 50 µl was denatured with Laemli buffer and heated at 95ºC for 10 min. Cells were lysed with RIPA buffer (50 mM Tris HCl pH. 8, 150 mM NaCl, 1% TritonX-100, 0.5% sodium deoxycholate, 0.1% SDS) containing protease/phosphatase inhibitor cocktail (Cell Signaling, #5872, Danvers, MA, USA) for cell lysis for 20 min. We then centrifuged the cell extract at 15,000 g at 4ºC for 20 min and collected cell lysate. Protein concentration in the lysate was assessed by a Micro BCA Protein Assay Kit (Thermo Fisher, Reinach, Switzerland). We used home-made Tris–glycine gels in Tris–Glycine buffer as previously described [40] using the following primary and secondary antibodies: exosomal marker antibody sampler Kit (Cell Signaling, Danvers, MA, USA, #74220), Calnexin (Cell Signaling 26793). All primary antibodies were diluted in a TBST buffer containing 5% bovine serum albumin. The incubation was performed at 4ºC overnight or for 1.5 h at room temperature. For secondary antibodies, we used anti-rabbit and anti-mouse IgG, HRP-linked antibodies (Cell signaling #7074 and #7076) using 1:2000 dilution in 1% milk TBST for 1 h incubation. The detection of chemiluminescent signal (Clarity Western ECL Western blot substrate, Biorad, Cressier, Switzerland) was conducted using chemiluminescent appliance (Fusion-FX7.826, program FusionCapt).

### RNase treatment of EVs

We isolated EVs using the supernatant of PM-E1 and non-PM-1 cells, using serum free medium as a negative control. 0.5 μg of RNA isolated from rat cells expressing luciferase [41] were added to all samples as a spike-in free RNA positive control. Then, RNase A (Qiagen) was added to the final concentration of 20 µg/ml. All samples were further incubated at 37 °C for 20 min, followed by RNA isolation and RT-qPCR.

### RNA isolation and sequencing

Total RNA of EVs and cells was isolated using mirVana PARIS extraction kit (Thermo Fisher). 500 µl of EV eluate was mixed with 500 µl 2X denaturing solution and 20 µl of 5 mg/ml Glycogen (AM9510, ThermoFisher Scientific, Zurich, Switzerland). Cell pellet was lysed with 400 µl ice-cold cell disruption buffer and mixed with an equal volume of 2X denaturing solution. Afterwards, the RNA isolation was performed according to the manufacturer’s instructions. 85 µl of the RNA solution was obtained from this kit. RNA concentration was measured using a Qubit RNA High sensitivity kit (Q32852, ThermoFisher Scientific). For library preparation, 20 ng/µl of RNA solution was prepared in water and subjected to library preparation with Illumina Truseq Total RNA kit (ribosomal depletion) according to the manufacturer’s instructions. RNA was sequenced using the Illumina Novaseq 6000 #1 platform to obtain 101 bp single-end reads at the Functional Genomics Center Zurich. To concentrate RNA for the analysis by RT-qPCR, the RNA samples (85 µl) were further precipitated using sodium acetate and resuspended in a total volume of 15 µl.

### RNA-seq data analysis

Quality of raw fastq files was analyzed using FastQC (https://www.bioinformatics.babraham.ac.uk/projects/fastqc/; version 0.11.9). Sequencing adapters were trimmed using Seqtk toolkit (https://github.com/lh3/seqtk; version 1.3-r117) and reads with quality below 20 were filtered out using Trimmomatic (http://www.usadellab.org/cms/?page=trimmomatic; version 0.39). Processed reads were mapped to the human reference genome GRCh38.p13 using STAR (https://github.com/alexdobin/STAR; version 2.7.5c). Gene expression was counted using Kallisto (https://pachterlab.github.io/kallisto/download.html; version 0.46.1) and gencode GTF annotation file (https://www.gencodegenes.org/; version 35). Data from the 4 sequenced samples was compared with publicly available RNA-seq data from 32 healthy plasma EV samples. Raw fastq files were downloaded from Gene Expression Omnibus (https://www.ncbi.nlm.nih.gov/geo/; GSE100206) and analyzed analogously as pleural mesothelioma samples. Given that the analysis involved two different data sets, batch effect correction was performed using the Qsmooth R package (https://bioconductor.org/packages/release/bioc/html/qsmooth.html). Further, genes upregulated in PM-EV samples were identified using differential gene expression analysis with edgeR R package. For the biotype analysis of expressed genes, the gencode GTF file (version 35, as above) was used. The longest transcripts based on the gencode annotation file were used for the comparison of transcript length and the number of exons of the PM-EV genes. Gene set enrichment of the upregulated PM-EV genes was calculated using hallmarks of cancer from MsigDB (https://www.gsea-msigdb.org/gsea/msigdb/) and fgsea R package (https://bioconductor.org/packages/release/bioc/html/fgsea.html). Annotation of transcriptional factors was obtained from the FANTOM5 database (https://fantom.gsc.riken.jp/5/). *S*- and *E-*component marker genes were obtained from [8].

### Survival analysis

RSEM-normalized gene expression from the The Cancer Genome Atlas (TCGA) mesothelioma (MESO) cohort, along with associated clinical information, was utilized for Cox regression analysis. Specifically, Cox survival analysis was performed individually for each selected and available gene, first using univariate models to assess the effect of gene expression alone and then using multivariate models that incorporated gene expression, patient age and disease stage. All analyses were run using the survival R package.

### RT-qPCR

We performed cDNA synthesis using 100–500 ng of starting material with PrimeScript RT and gDNA Eraser (TAKARA Bio, S-86901–06-01, Saint-Germain-en-Laye, France). The cDNA synthesis was conducted in a 20 µl reaction volume and subsequently diluted with 100 µl of water to a total volume of 120 µl. Three µl of the sample was used for quantitative real-time PCR (qPCR) in at least technical duplicates, using KAPA SYBR FAST qPCR master mix with low ROX (Kapa Biosystems, KK4621 Merck, Buchs, Switzerland) in a 10 µl reaction volume. For qPCR, we used 200 nM of each primer and performed the reaction on a 7500 fast real-time PCR system (Life Technology, Life Technology Europe BV, Zug, Switzerland) with the following conditions: 95ºC for 3 min for enzyme activation, followed by 95ºC for 3 s for denaturation, and 60ºC for 30 s for annealing/extension, for a total of 40 cycles. The specificity of the products was confirmed by the presence of a single product during melt curve analysis. No template control was used as a negative control for both cDNA synthesis and qPCR. We assumed a reaction efficiency of 100%. Gene expression was quantified using delta Ct and beta-actin as the reference gene for both EVs and cells. Primers used are listed in Table S8.

## Results

### Characteristics of EVs isolated by ExoEasy Maxi kit

**Fig. 1:**
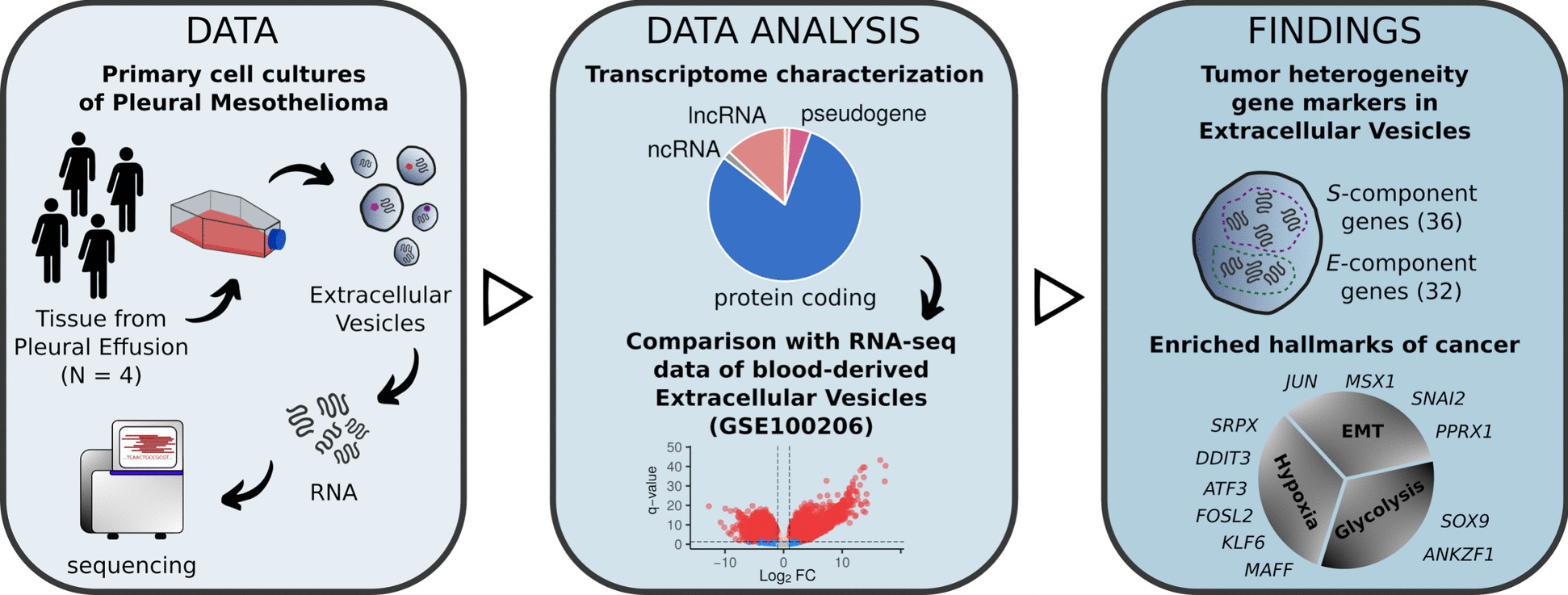
Study overview. Extracellular vesicles (EVs) were isolated from cell culture supernatant of four pleural mesothelioma (PM) cell cultures and profiled using RNA sequencing. The transcriptomic profiles of these EVs were compared to publicly available gene expression data from EVs isolated from 32 healthy plasma samples. Genes expressed in pleural mesothelioma-derived EVs (PM-EVs) were further linked to pleural mesothelioma-specific transcriptional compartments, and the enrichment of cancer hallmarks within PM-EVs was characterized

**Fig. 2:**
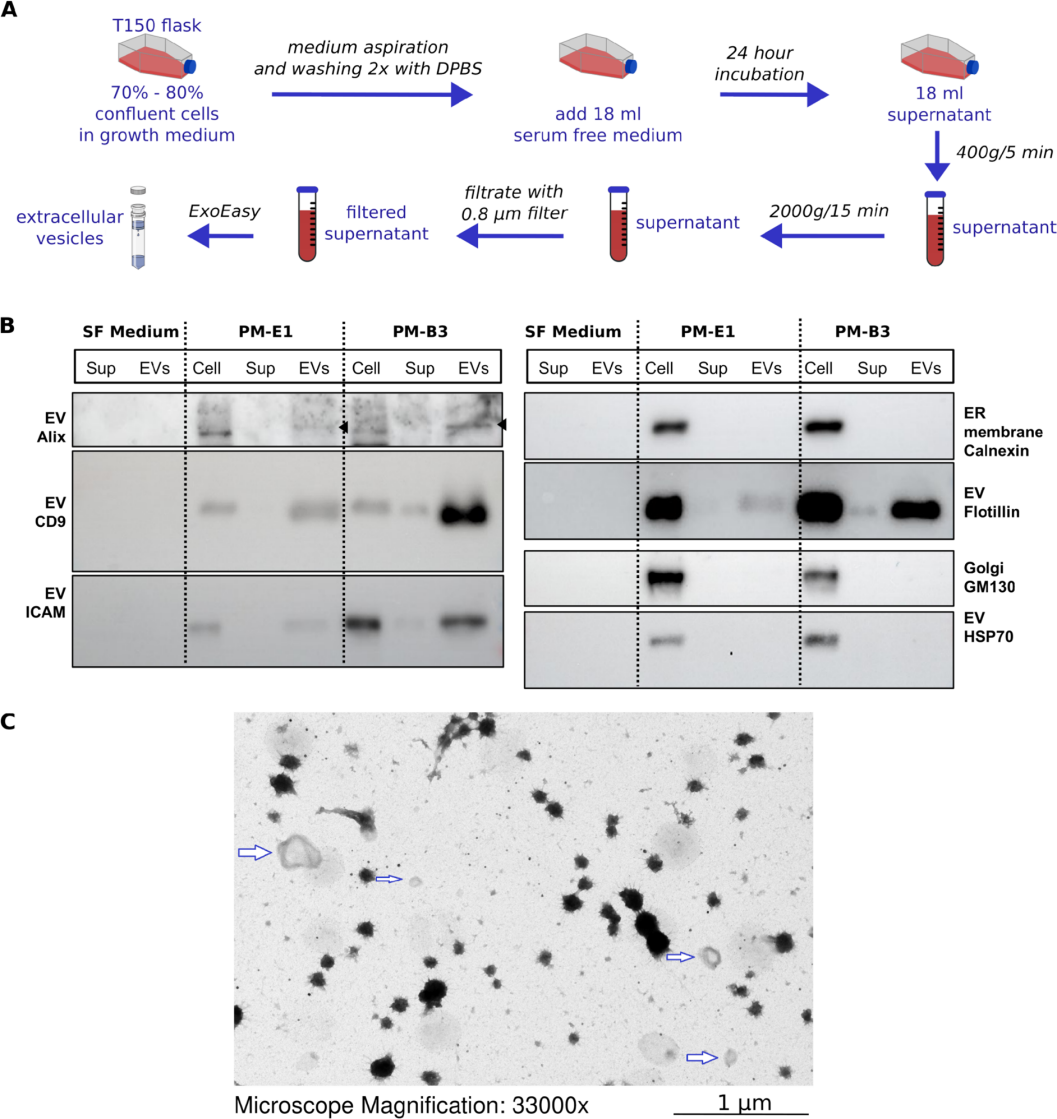
Isolation of extracellular vesicles secreted by pleural mesothelioma primary cells. **A** Experimental overview. **B** Western blot of EV-specific protein markers including Alix (cytosolic protein in EVs), CD9 (transmembrane protein), HSP70 (cytosolic protein in EVs), Flotillin (cytosolic protein in EVs with lipid or membrane binding capacity), CD54/ICAM (cell surface glycoprotein). We selected markers for potential contaminant organelles including endoplasmic reticulum transmembrane (Calnexin) and Golgi matrix (GM130). Antibodies were tested specifically in cells, cell culture supernatant (Sup) and in extracellular vesicles (EVs), using serum free (SF) medium without cells as negative control. We performed SDS-PAGE on the samples in triplicate, loading equal amounts of protein. Uncropped blots are provided in Additional File 4. **C** Electron microscopy of isolated EVs. Arrows indicate microvesicles and exosomes. Dark spots are the artifacts associated with the staining

To investigate the transcriptomic secretome of PM cells, we analyzed the RNA cargo of EVs isolated from cell culture supernatants derived from four PM patients (Figs. 1 and 2A). Clinical information associated with the matching PM tumors is included in Table 1.

The identification of PM cells was achieved using immunohistochemical staining of epithelial and mesothelial markers including pan-Cytokeratin, Podoplanin, Calretinin, Wilms’ Tumor 1 (WT1) and BRCA1 Associated Deubiquitinase 1 (BAP1) staining (Supplementary Figure S2). While we observed down regulation of Calretinin in all primary cells compared to the tumor tissues, the remaining markers for mesothelial cell origin, including Podoplanin and WT1, remained positive. More than 99% of these cells were positive for pan-Cytokeratin, effectively ruling out contamination by non-epithelial cells, particularly fibroblasts and macrophages. We found that BAP1 staining in cells corresponded to tumor tissues. Specifically, PM-E1 and PM-B1 were positive for nuclear BAP1 while the remaining cell lines were weak-negative. In addition, to ensure that we were profiling malignant cells, we used Copy Number Arrays to verify whether the copy number alteration profiles of the grown primary cells matched those of the original tissues. Specifically, we confirmed that the profiles corresponded to expected pleural mesothelioma-specific alterations, including changes in the *BAP1* and *MTAP*/*CDKN2A* gene regions (Additional File 5).

To investigate the enrichment and purity of collected vesicles, we used western blotting to detect known EV markers (Fig. 2B) [39]. We tested the expression of EV-enriched proteins, including Alix (cytosolic protein in EVs), CD9 (transmembrane protein), HSP70 (cytosolic protein in EVs), Flotillin (cytosolic protein in EVs with lipid or membrane binding capacity), CD54/ICAM (cell surface glycoprotein) in two of the analyzed PM cell lines. For each of them, the expression of the markers was tested in three fractions, including cells, supernatant, and isolated EVs. To ensure the specificity of isolated EVs, we selected contamination markers associated with the Golgi apparatus matrix (GM130) and the endoplasmic reticulum membrane (Calnexin). All EV markers, except for cytosolic protein HSP70, were detectable in both cells and EVs. The absence of HSP70 in EVs is likely attributable to its lower abundance in these vesicles. However, another cytosolic protein, Alix, was detectable in the EV fraction, further validating the enrichment of EVs achieved with the used preparation technique. As expected, GM130 and Calnexin were observed in the cells and absent in supernatants and EVs (Fig. 2B), supporting the absence of contaminants and apoptotic bodies.

While attempting to identify the concentration and size of EVs by nanoparticle tracking analysis (NTA), we observed that the elution of the ExoEasy kit, even without sample loading, generated a high concentration of particles ranging from 50–300 nm (data not shown). These particles have the same size range as EVs, thereby limiting the feasibility of NTA for EV characterization. Simultaneously, we used electron microscopy to verify the morphology of isolated EVs. While it was proven that EVs isolated with the ExoEasy kit were difficult to visualize with this technique since the EVs in the elution buffer do not attach well on the slides [42], we managed to capture some of the EVs with this method (Fig. 2C). Obtained images confirmed a round shape membrane of the vesicles (Fig. 2C). Altogether, these observations confirmed that we could isolate a pure and diverse population of EVs using the ExoEasy kit.

### Pleural mesothelioma cells secrete extracellular vesicles enriched with protein-coding and long non-coding RNAs

**Fig. 3:**
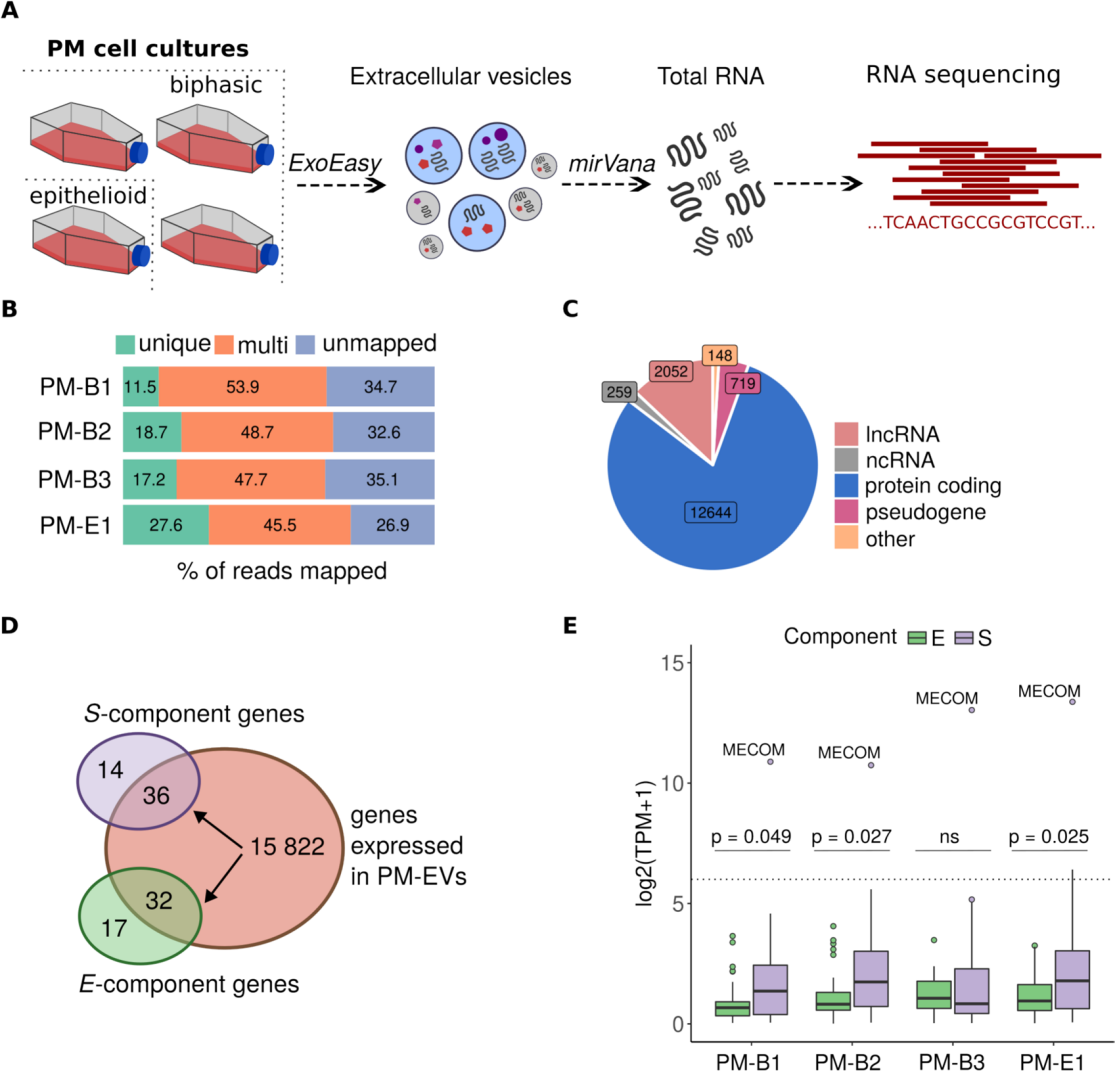
Transcriptomic characterization of PM-EVs (*n* = 4). **A** Overview of RNA isolation from EVs. **B** Mapping rate of the RNA-seq reads from the 4 PM-EV samples to the human genome. **C** Distribution of biotypes of the genes expressed across the 4 PM-EV samples. lncRNA: long non-coding RNA, ncRNA: non-coding RNA. **D** Overlap between *S-* and *E-component* marker genes identified in PM cell lines and genes expressed across the 4 PM-EV samples. **E** Expression of the *S-* and *E-component* genes across the 4 PM-EV samples. The dotted line represents the median expression of all genes expressed across the analyzed samples. TPM: transcripts per million

In the next steps, we isolated total RNA from collected PM-EV samples and performed RNA sequencing (Fig. 3A). Electrophoresis analysis of the extracted RNA revealed an enrichment of RNA molecules below 200 nucleotides in size, and the absence of ribosomal RNAs (Supplementary Figure S1). As we were interested in profiling the whole transcriptomic cargo of EVs secreted by PM cells, we sequenced total RNA to be able to detect coding and non-coding RNA populations. After sequencing, reads were preprocessed and mapped to the human reference genome (Fig. 3B, Table S1). The obtained mapping rate highlighted a high proportion of reads mapping to the genome, which confirms that PM-EVs contain RNA that represents known human genes.

Given a high proportion of multimapping reads, we used Kallisto to calculate gene expression for the downstream analysis. Kallisto allows for estimating gene expression by probabilistic assignment of multimapping reads to genes, allowing us to recover reads which would be removed in the classical approach. Next, we were interested in the distribution of biotypes of expressed genes. Our analysis revealed that the majority of expressed genes were protein-coding genes (80%), long non-coding RNAs (13%) and pseudogenes (4.5%), with a low proportion of short non-coding RNAs (1.6%) (Fig. 3C, Table S2). Interestingly, we observed a lower total number of expressed genes in EVs compared to the expected number of genes expressed in cells, which may suggest a selective sorting of RNA cargo into vesicles.

### Pleural mesothelioma extracellular vesicles contain genes associated with *S-/E-*phenotypes

Given the potential role of EVs in treatment and disease detection, we wanted to check if EVs secreted by PM cell lines harbor genes associated with intratumor heterogeneity. Therefore, we used previously published marker genes of *S-* and *E-*compartments specific for PM cells. Within the 15,786 genes expressed across 4 PM-EV samples, we detected 36 and 32 genes from the *S-* and *E-*component signatures, respectively (Fig. 3D, Table S3). Because the *S-* and *E-*components were shown to be expressed even within cells of epithelioid origin [8–10], we wanted to verify if these markers were also detectable within PM-EVs secreted by the epithelioid cells. Specifically, we calculated scores of the expressed *S-* and *E-* marker genes as a sum of the expression of these markers in each PM-EV sample (Fig. 3E). Interestingly, we observed a consistent pattern of expression of both *S-* and *E-* markers even within the epithelioid sample. In three out of four samples, the *S-*markers had significantly higher expression than the *E-*markers. Moreover, *MECOM*, a gene encoding a transcription factor and one of the *S-*component marker genes, was highly expressed across all samples. *MECOM* has been previously linked with cancer stem cell properties [43], malignant transformation [44, 45], cell proliferation and inhibition of apoptosis through the AKT/mTOR pathway. Given the consistent high expression of this gene in PM-EVs across both histological subtypes, we hypothesize that its elevated secretion may be linked to more advanced disease, as three out of the four original tumors were stage 3 and all tumors showed lymph node metastases (Table 1). However, further research is needed to understand the functional role of this gene in both donor and recipient cells.

### PM-EVs RNA cargo present different characteristics than the RNA cargo of EVs isolated from healthy plasma

**Fig. 4:**
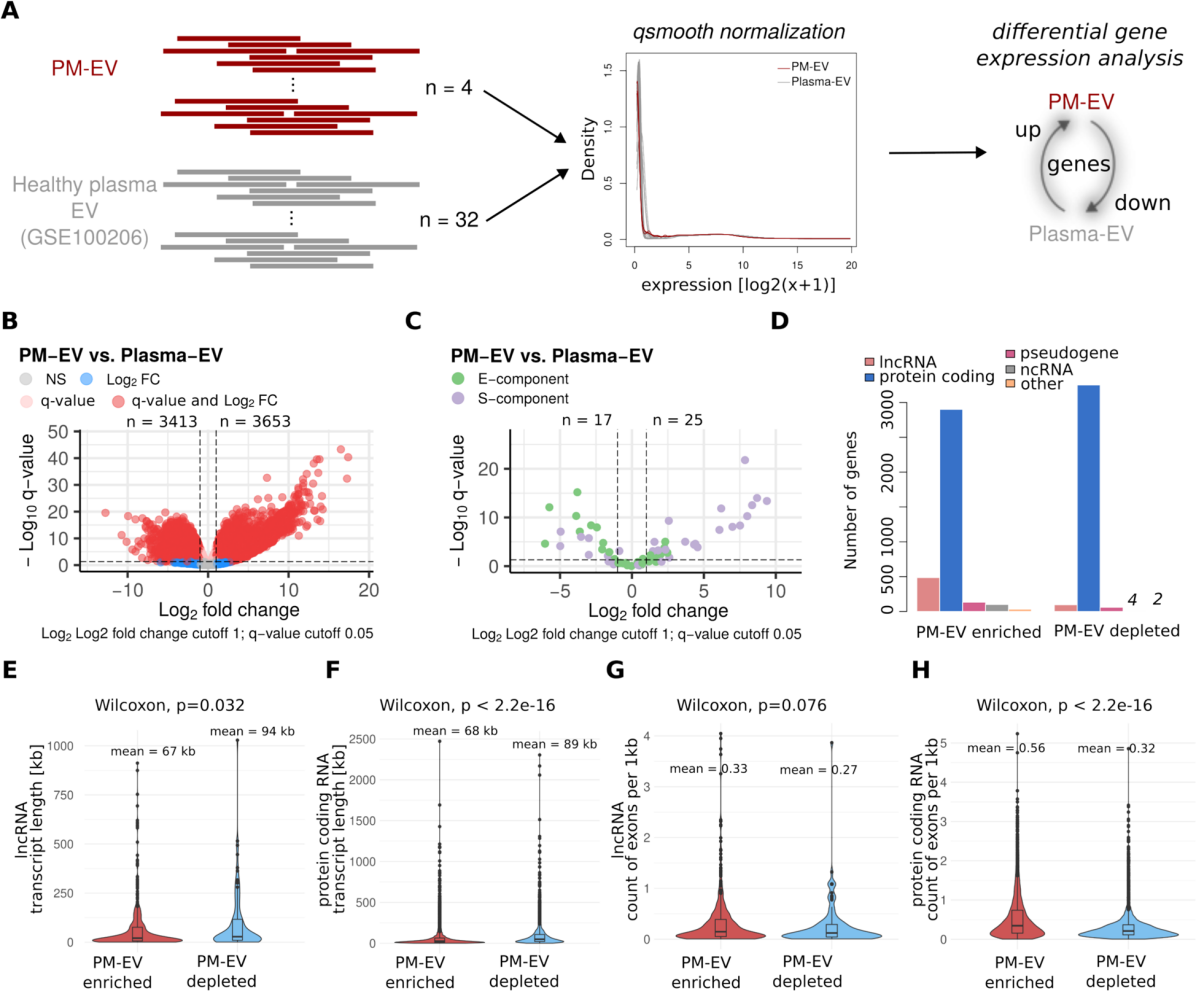
Characterization of PM-EV deregulated genes. **A** Overview of computational analysis aimed at comparing PM-EV and Plasma-EV gene expression. Raw fastq files were processed using the same pipeline, gene expression was counted using Kallisto, followed by *qsmooth* normalization of the two datasets, and differential gene expression. **B** Genes differentially expressed between PM-EV and Plasma-EV samples. Points are colored according to passed filters. NS—not significant. **C** *S-* and *E-component* marker genes are differentially expressed between PM-EV and Plasma-EV samples. **D** Gene biotypes among PM-EV enriched and depleted genes. **E–H** Violin plots of long non-coding RNA (**E** and **G)** and protein-coding RNA (**F** and **H**) presenting distribution of transcript length (**E** and **F**) and number of exons (**G** and **H**) among PM-EV enriched and depleted genes

In order to better characterize RNAs secreted into PM-EVs, we used publicly available RNA-sequencing data from extracellular vesicles isolated by the ExoRNeasy kit from healthy plasma samples (Fig. 4A, Table S9). In short, raw fastq files were processed analogously as PM-EV data, and after gene expression quantification, both sets of samples were normalized together using *qsmooth* normalization to remove batch effects (Table S4). We then used differential gene expression analysis to identify up- and downregulated genes between the two conditions. Specifically, we found 3,413 and 3,653 genes to be down- and upregulated in PM-EV compared to Plasma-EV samples (Fig. 4B, Table S5). Among both of the gene groups, the majority consisted of protein-coding genes, however, a substantially high number of long non-coding RNAs was enriched in PM-EVs (Fig. 4D). Considering the role of long non-coding RNAs in regulating gene expression, cell proliferation and apoptosis, previous studies linked their expression with cancer invasion and progression [46]. In light of that, long non-coding RNAs enriched in PM-EVs present a potentially interesting population of functionally relevant molecules for diagnosis and treatment of the disease. Additionally, we observed 25 *S-* and *E-*marker genes to be enriched in PM-EVs compared to Plasma-EVs (Fig. 4C). While a subset of the heterogeneity-linked marker genes was not significantly upregulated in our samples, the ones significantly upregulated may be transported to recipient cells and initiate phenotypic changes. Overall, these results underline that vesicles secreted by PM cells contain RNA that could play an important role in remodeling recipient cell transcriptomes.

As long non-coding RNA and protein-coding genes were the most prevalent RNAs dysregulated in PM-EVs, we next verified whether transcripts associated with these genes harbor specific properties. Both in the case of long non-coding RNAs and protein-coding genes, the transcripts enriched in PM-EVs were consistently shorter than those enriched in Plasma-EVs (Fig. 4E and 4F). At the same time, the PM-EV enriched transcripts had more exons per one kilobase compared to the Plasma-EV enriched ones (Fig. 4G and 4H). These features have been previously linked with increased RNA stability [47, 48], which underlines the hypothesis that PM-secreted vesicles contain stable and functional RNA.

### Expression of PM-EV enriched genes correlates with cellular gene abundance

**Fig. 5:**
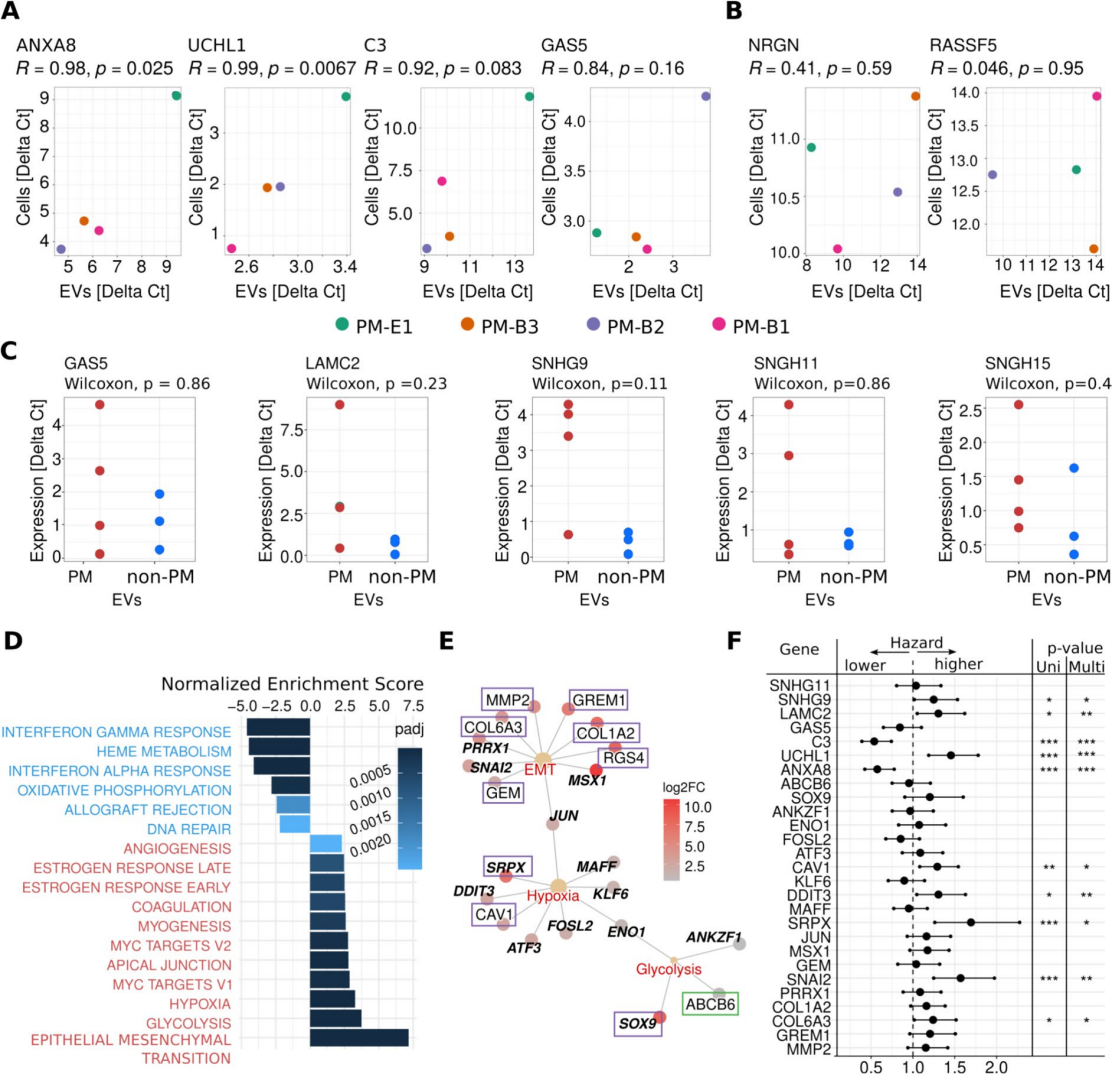
PM-EV upregulated genes are expressed in donor cells and linked with hallmarks of cancer and patient survival. **A-B** Quantitative PCR analysis of selected PM-EV enriched (**A**) and depleted (**B**) genes in EVs and matching PM cells. Pearson’s R and p-value are reported for each gene. **C** Quantitative PCR analysis of expression of PM-EV enriched genes in PM-EVs and non-PM-EVs. Wilcoxon’s test p-value is reported. **D** Gene set enrichment analysis of hallmarks of cancer in PM-EV upregulated genes. Pathways with positive and negative normalized enrichment scores, along with adjusted p-values, are shown in red and blue, respectively. **E** Transcriptional factors (in bold) and *S-* (in orchid) and *E-* (in green) marker genes most associated with EMT, hypoxia, and glycolysis in PM-EV samples. Dots are colored according to log2 fold change of the enrichment in PM-EV compared to Plasma-EV samples. **F** Cox regression survival analysis using selected PM-EV enriched genes in TCGA MESO cohort. Significant *p*-value (< 0.05) from univariate (Uni) and multivariate (Multi) models is reported. ***: *p* < 0.001, **: *p* < 0.01, *: *p* < 0.05

The relationship between the abundance of RNAs in donor cells and secreted vesicles has not been fully explored yet. While some RNA molecules can be present in both, other RNAs can be preferentially secreted to be eliminated from donor cells. To verify that the upregulated genes are produced by donor cells, we selected four genes highly enriched in PM-EVs—*ANXA8*, *UCHL1*, *C3* and *GAS5*—and verified their expression in matching PM cells using RT-qPCR. The expression patterns of the genes in PM-EVs were consistent with their expression in matching cells (Fig. 5A, Table S6). Then, we analyzed two genes, *NRGN* and *RASSF5*, which were significantly depleted in PM-EVs, and found a much weaker association between their expression in EVs and matching cells (Fig. 5B, Table S6).

### PM-EV biomarker candidates show promise for clinical application

We also sought to determine whether the expression of the identified enriched genes is higher in EVs isolated from PM cells compared to non-malignant mesothelial (non-PM) cells, which would further support the clinical relevance of the candidate biomarkers. Given the role of lncRNAs in cancer progression and their high enrichment in PM-EVs, we selected four lncRNA genes: *GAS5*, *SNHG9*, *SNHG11* and *SNHG15*, along with *LAMC2*—a protein coding gene, whose high expression has been associated with metastasis and poor prognosis in some cancers including PM [49]—for additional validation using EVs isolated from three non-PM cell cultures (Table S10). The non-PM cells were derived from pleural effusion of patients without malignancy. Similarly as for the PM cell cultures, the identity of non-PM cells was confirmed using staining for epithelial and mesothelial markers pan-Cytokeratin, Podoplanin and WT1 (Supplementary Figure S2), as well as Copy Number Array analysis. In contrast to PM cells, non-PM cells presented normal chromosomal profiles (Additional File 5).

The RT-qPCR analysis of selected markers in PM-EVs and non-PM-EVs revealed a trend toward higher expression in PM-EVs (Fig. 5C, Table S11). However, further validation with additional samples is required to confirm the elevated expression of these markers in PM-EVs.

Next, we tested the expression of these five markers in EVs isolated from plasma samples of four PM patients. The plasma samples were used to evaluate the potential of these genes as blood-based biomarkers of PM. Although detected at low levels, *SNHG9*, *GAS5* and the loading control beta-Actin were detectable in EVs isolated from PM-plasma specimens of all four patients (mean Ct: 35.5, 30.9 and 27.2, respectively). These results suggest that markers enriched in PM-EVs are detectable in clinical samples and hold potential as circulating biomarkers. However, additional validation using additional plasma samples is necessary to fully verify their clinical applicability.

To confirm that the tested secreted RNA is encapsulated in EVs, we treated EVs with RNase using two representative cell cultures, PM-E1 and non-PM-1. The RNA degradation was assessed by comparing the Ct values (Delta Ct) between RNase-treated and untreated samples (Ct_treated_—Ct_untreated_). While strong degradation of the spike-in free RNA control (luciferase) was observed (Delta Ct: 14.6 for PM-E1 and 15.7 for non-PM-1), one of the biomarkers, *GAS5*, showed significantly less degradation (Delta Ct: 4.2 for PM-E1 and 5.3 for non-PM-1). The other four biomarkers, expressed at lower levels in the treatment-naive samples, showed no detectable signal after RNase treatment. While some degradation was observed, *GAS5* expression was much more stable in EVs compared to free RNA. While these results suggest that some of the biomarkers may not be entirely within the luminal part of EVs, the degradation could also be attributed to RNase carryover, causing additional RNA degradation in the downstream analysis.

### PM-EVs carry RNA cargo associated with hallmarks of cancer

To further characterize the molecular function of upregulated PM-EV genes, we analyzed the enrichment of genes associated with hallmarks of cancer within the set of PM-EV upregulated genes. The analysis revealed that PM-EV upregulated genes were significantly associated with several known oncogenic processes (Fig. 5B, Table S7). Among the top enriched hallmarks were Epithelial-to-Mesenchymal Transition (EMT), glycolysis, and hypoxia, as well as processes linked to cell proliferation (i.e., MYC targets). Interestingly, several molecular processes typically upregulated in PM, such as interferon response, oxidative phosphorylation and DNA repair [8], were significantly depleted in PM-EVs. Several PM-EV enriched genes are particularly interesting due to their functional roles in cancer progression. *MMP2*, *COL6A3*, *PRRX1*, *COL1A2*, *GREM1* and *SNAI2* are integral to the EMT process, a key driver of metastasis that allows tumor cells to detach and invade distant tissues. Notably, *COL6A3* and *PRRX1* have been shown to confer resistance to chemotherapeutic agents like cisplatin [50, 51], and circulating *COL6A3* has been proposed as a prognostic biomarker for colorectal and breast cancers [52, 53]. Among other upregulated genes, *CAV1* was shown to be involved in EV formation and cargo sorting [54], particularly within hypoxic TME in gastric cancer [55]. Glycolysis-regulating genes such as *SOX9*, *ABCB6* and *ENO1*, were associated with metabolic reprogramming essential for cancer progression [56, 57]. For instance, *ENO1* supports tumor survival in nutrient-deprived TME by promoting angiogenesis and sustaining glycolytic activity [58, 59]. Similarly, *SOX9*, a master regulator, promotes proliferation and metastasis [60–62], as well as ATP-driven invasion and chemoresistance [63].

Further, we identified transcription factors and *S-* and *E-s*ignature genes that had the strongest association with the top three enriched hallmarks (Fig. 5E, Table S7). As expected, the EMT-linked genes included multiple *S-*component genes, which is particularly significant given their association with worse patient prognosis [8]. Together, these results support the hypothesis that genes secreted through EVs from pleural mesothelioma cancer cells are functionally active and linked with pro-oncogenic phenotypes as well as hold potential as EV-based circulating biomarkers for PM detection.

Finally, to determine the prognostic relevance of the selected genes associated with top enriched hallmarks as well as the qPCR-validated candidates, we analyzed RNA-seq and clinical data from The Cancer Genome Atlas (TCGA) pleural mesothelioma cohort. Eight genes (*CAV1*, *DDIT3*, *SNAI2*, *LAMC2*, *COL6A3, UCHL1, SRPX* and *SNHG9*) were significantly associated with worse patient survival based on their expression in tissue samples (Cox regression analysis, p-value < 0.05; Fig. 5F). These genes were predictive of survival both in univariate analyses and in multivariate models which included patient age and disease stage (Fig. 5F). Together, these findings highlight the potential of PM-EV enriched genes as prognostic biomarkers for pleural mesothelioma, linking their expression to patient outcomes.

## Discussion

We conducted a thorough characterization of the RNA cargo of extracellular vesicles (EVs) secreted by pleural mesothelioma (PM) primary cells. We found that PM cells secrete a heterogeneous population of vesicles, most probably composed of microvesicles and exosomes. These vesicles contain a variety of RNA populations, predominantly protein-coding and long non-coding RNAs. This discovery is crucial for advancing our understanding of cellular dynamics and communication within the tumor microenvironment, especially given recent evidence that secreted RNAs retain their regulatory functions in recipient cells and can be translated into functional molecules [64–66]. Long non-coding RNAs, such as *GAS5*, are particularly interesting due to their significant roles in carcinogenesis [46] and modulating tumor microenvironment [67]. For instance, *GAS5* has been shown to influence macrophage and T-cell infiltration in non-small cell lung cancer [68] and has been implicated as a circulating diagnostic biomarker in plasma of pleural mesothelioma patients [69]. *GAS5* is associated with cellular quiescence and podoplanin expression in vivo, further highlighting its relevance to PM biology [70]. Additionally, we identified other lncRNAs enriched in PM-EVs, such as *SNHG9*, *SNHG11* and *SNHG15*, which have been previously associated with cancer development [71–78] and were associated with mesothelioma patients’ survival in our analysis. These findings emphasize the promise of lncRNAs as both diagnostic biomarkers and therapeutic targets, particularly given advancements in antisense oligonucleotide and small interfering RNA-based approaches to silence oncogenic lncRNAs [79, 80]. Identifying potential long non-coding RNA targets in mesothelioma brings us closer to applying these emerging therapies, thereby expanding the range of available treatments for this disease.

Interestingly, PM-EVs carry previously reported gene markers associated with intratumor heterogeneity of PM cells, characterized by epithelioid (*E*) and sarcomatoid (*S*) marker genes [8]. Surprisingly, although the PM cells originate from pleural effusion (PE), which is generally reported to not contain sarcomatoid cells [81], we observed a similar expression pattern of the *E-* and *S-* marker genes across all 4 PM-EV samples, with significantly higher expression of the *S-*markers even in the EVs secreted by the cells from the epithelioid tumor. One explanation could be that epithelial cells shed into PE may present an enrichment of *S-component* gene signatures, despite maintaining an epithelial morphology. While the epithelioid classification of the PM-E1 cells comes from the histological evaluation of matched tissue samples, the transcriptomic profile of the mesothelial cells from the tumor may be more enriched with the *S-*phenotype genes. As a result, the higher expression of these genes may be related to their increased secretion by donor cells. This could be due to several mechanisms: cells might expel these genes to mitigate potentially harmful effects on their cancerous phenotype or because these genes are no longer required. Alternatively, elevated secretion might represent a targeted mechanism for transferring functionally active genes involved in oncogenic processes, as seen in other cancer types [32–36]. Indeed, the tumors associated with the PM-EV samples were mostly linked to more advanced disease and lymph node metastases, but to explore this relationship in more detail, additional samples from early-stage tumors are necessary. Conversely, genes essential for maintaining donor cell function may be selectively retained, which could account for the observed downregulation of hallmark pathways typically enriched in PM, such as DNA repair, interferon response and oxidative phosphorylation, within PM-EVs [82]. This observation aligns with our findings from the comparison of specific marker expression between EVs and their corresponding donor cells, but broader research is needed to elucidate the mechanisms driving gene secretion and to evaluate the functional roles of these genes in both donor and recipient cells.

The presence of markers associated with the *S-component* suggests that PM-EVs may carry genes linked with a more aggressive tumor phenotype and worse patient prognosis [8]. The *S-component* has been implicated in the infiltration of T-cells, monocytes, fibroblasts and endothelial cells, promoting angiogenesis in PM tumors [8]. This result highlights the potential role of PM-EVs carrying *S-component* markers as critical players in remodeling the TME of PM tumors. Finally, the presence of transcriptional factors, such as *SNAI2*, *SRPX*, and *DDIT3*, whose expression in PM tumors is linked with patient survival based on our analysis, further supports the role of PM-EVs in modulating TME-resident cells.

When comparing RNAs expressed in PM-EVs with those expressed in EVs isolated from healthy plasma samples, we found a big variation in up- and downregulated protein-coding genes and a noticeably higher number of upregulated long non-coding RNAs. The PM-EV enriched mRNA and long non-coding RNA transcripts were shorter and contained more exons than the PM-EV depleted ones, highlighting their properties linked with a more stable form of RNA. This evidence suggests that EV-secreted transcripts could be selectively sorted and packed to ensure the high stability of carried RNA to maintain their biological activity until delivered to the recipient cells.

Finally, our results underline the clinical potential of PM-EV cargo genes. Several genes enriched in PM-EVs, such as *COL6A3*, *CAV1*, *SRPX* and *DDIT3*, are associated with key oncogenic processes like EMT, glycolysis and hypoxia. These genes hold promise as circulating biomarkers for PM, with applications ranging from disease detection to assessing patient prognosis as revealed by our analysis using TCGA data. For example, *LAMC2*, which drives EMT and metastasis in lung adenocarcinoma [83], and *ANXA8*, which is upregulated in various cancers including PM [84–87], could serve as important diagnostic tools. Similarly, *UCHL1* has increased expression in tissues and plasma from patients with neuroendocrine carcinomas [88], while tumor-derived *C3a* expression was elevated in serum samples of patients across multiple cancer types [89–91]. Furthermore, current research into the regulation of EV biogenesis and secretion is uncovering potential applications for cancer therapies [92, 93]. Consequently, vesicles containing these genes may emerge as novel therapeutic targets in the future, although additional studies are required.

Our study faces several limitations. Firstly, the sample size of analyzed pleural mesothelioma samples is relatively small, potentially limiting the validity of our findings and leading to sample-specific discoveries. Moreover, while our analyses encompass samples from epithelioid and biphasic tumors, the sarcomatoid subtype of mesothelioma is underrepresented in our comparison, which may constrain the scope of conclusions towards a comprehensive representation of PM secretome.

Secondly, our investigation into PM-EV cargo is based on the assumption that these vesicles are released into the bloodstream, a phenomenon observed in other cancer types, such as prostate cancer and melanoma [94, 95]. Despite being diluted by EVs from other cells, tumor-derived PM-EVs in plasma likely carry biomarkers that can be detected and utilized for disease diagnosis. To increase the sensitivity of discovering such biomarkers, we began by focusing on primary PM cells to identify genes highly enriched in EVs secreted by these cells. Then, to verify this hypothesis, we compared PM-EV RNA cargo with RNA composition of EVs isolated from healthy plasma samples. This comparison introduces a notable batch effect, as the samples originated from different sources (primary cell culture secretome vs. plasma), were generated in separate experiments conducted at different institutions, and show variation in clinical characteristics, particularly in gender and age distribution. Despite our efforts in batch normalization before comparison, the disparity may still confound our results and affect the accuracy of our conclusions. To mitigate this, we validated expression of a small subset of PM-EV enriched markers in matching PM cells and non-PM cells, as well as assessed the expression of these markers in plasma samples from PM patients. While our results are promising, extensive validation using a larger cohort of plasma samples from both healthy individuals and PM patients is necessary.

Moreover, we recognize the potential impact of the EV isolation method, particularly due to the EV size heterogeneity, on our analysis. While gradient-based ultracentrifugation remains the gold standard for isolating pure EV populations, we opted not to use this method due to its potential to damage vesicles [96–98] and the method’s time-consuming, low-throughput nature, which limits its clinical applicability [97]. Instead, we chose the ExoEasy Maxi kit, which provides a straightforward and cost-effective protocol. Even though alternative methods like size exclusion chromatography (SEC) offer high-purity vesicle isolation, our primary focus was on capturing a broader range of EVs to maximize biological signal and biomarker detection potential, particularly in plasma. This is especially important for future clinical applications, as the concentration of tumor-secreted EVs is likely diluted in plasma due to the presence of non-tumor-derived EVs. However, for future studies aiming to explore homogenous EV subpopulations, we believe that SEC methods should be considered.

Despite these limitations, our study provides valuable insights into the field and identifies areas for further research and refinement. Although there is growing interest in harnessing RNAs within EVs as biomarkers and therapeutic targets in some cancers [35], similar research in PM remains underexplored. Our work is pivotal in addressing this gap, providing new understanding of the role of EVs and their RNA content in mesothelioma. By advancing this knowledge, we lay the groundwork for developing innovative diagnostic and therapeutic strategies, which could significantly enhance treatment options and outcomes for patients facing this devastating disease.

## Conclusions

Our work provides the first overview of the transcriptomic cargo of extracellular vesicles secreted by pleural mesothelioma cells. Our study characterizes the biotypes of RNA cargo, highlights its functional role in oncogenesis and pleural mesothelioma heterogeneity, and underlines EVs’ potential as a source for diagnostic biomarkers of this devastating disease.

## Supplementary Information

Additional File 1: Supporting data required to run the analysis, including S- and E-component signature genes from (8), number of exons in transcripts and transcript length.

Additional File 2: File containing R code used to perform the analysis.

Additional File 3: Commands used to process raw fastq files.

Additional File 4: Uncropped western blot images of EV markers.

Additional File 5: Copy Number Analysis of PM and non-PM cells.

Additional file 6: Table S1. Mapping rate of RNA-seq reads from PM-EV samples. Mapped reads were obtained with STAR. Table S2. Biotypes of genes expressed among 4 PM-EV samples. Expressed genes are those with Kallisto counts > 0 in all 4 samples. Table S3. TPM expression of the genes expressed across 4 PM-EV samples. TPM values provided by Kallisto. Table S4. Qsmooth batch-corrected and TMM normalized counts of Plasma-EV and PM-EV samples. Table S5. EdgeR results of differential gene expression between PM-EV and Plasma-EV samples. S and E marker genes are reported in the “marker” column. Table S6. Results of qPCR analysis of selected genes in PM-EVs and matching primary cells. Table S7. Results of fgsea enrichment analysis in PM-EV samples. Table S8. Sequences of primers used for qPCR analysis. Table S9. Characteristics of healthy plasma donors from GSE100206 dataset. Table S10. Clinical characteristics associated with the non-PM cells used for qPCR analysis. Table S11. Results of qPCR analysis of selected genes in PM-EVs and non-PM-EVs.

Additional file 7: Figure S1. Electrophoresis analysis of RNA extracted from pleural mesothelioma extracellular vesicles.

Additional file 8: Figure S2. Immunohistochemistry staining of PM and non-PM cells.

## Data Availability

All data supporting the findings of this study are available within the article and within its supplementary files. Raw sequencing data of EVs from plasma samples from healthy donors is available in GEO (GSE100206). The Cancer Genome Atlas mesothelioma RSEM RNA-seq data is available at UCSC Xena browser (https://xenabrowser.net/). The clinical data of the same dataset is available at https://gdc.cancer.gov/about-data/publications/pancanatlas.

## Abbreviations

PM: Pleural mesothelioma
EV: Extracellular vesicle
PM-EV: Extracellular vesicle secreted by pleural mesothelioma primary culture
E: Epithelioid
S: Sarcomatoid
NTA: Nanoparticle Tracking Analysis
Plasma-EV: Extracellular vesicle circulating in plasma of healthy donors
PCR: Polymerase chain reaction
EMT: Epithelial-Mesenchymal transition
mRNA: Messenger RNA
lncRNA: Long non-coding RNA
ncRNA: Non-coding RNA
TPM: Transcripts per million
TCGA MESO: The Cancer Genome Atlas pleural mesothelioma
SEC: Size exclusion chromatography

## Declarations

### Code availability

R code used to analyze the data have been provided in the Supplementary Files.
