# Supplementary material for: Exploring RNA cargo in extracellular vesicles for pleural mesothelioma detection
Source: BMC Cancer. 2025 Feb 7;25:212. doi: 10.1186/s12885-025-13617-y (PMC11804012; doi:10.1186/s12885-025-13617-y)
Supplement: Supplementary file 4 — Additional File 4: Uncropped western blot images of EV markers. [file 12885_2025_13617_MOESM4_ESM.pdf]

Additional File 4.pdf: Uncropped western blot images of EV markers.

Uncropped blots of figure 1B Blot 1 (left)

Alix

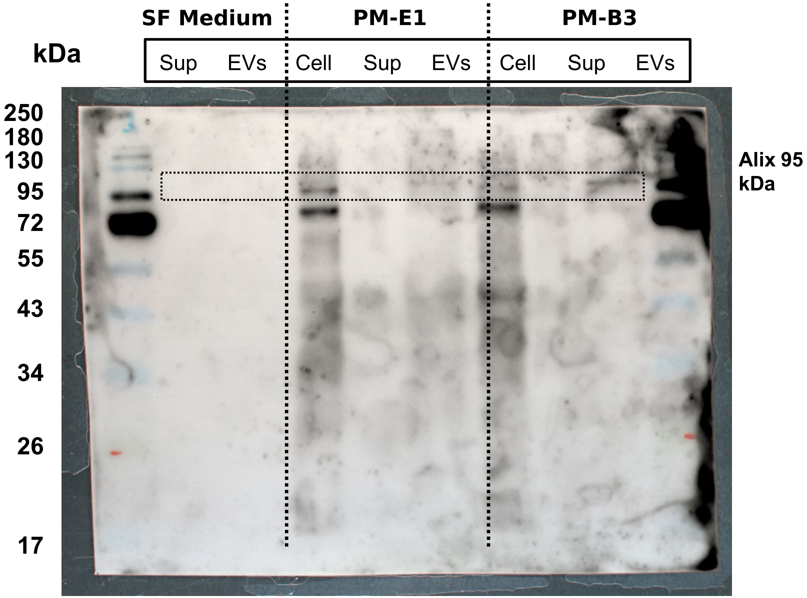

Uncropped blot merged with protein ladder
Fresh blot was stained with anti-Alix, exposure time 5 minutes.

CD9

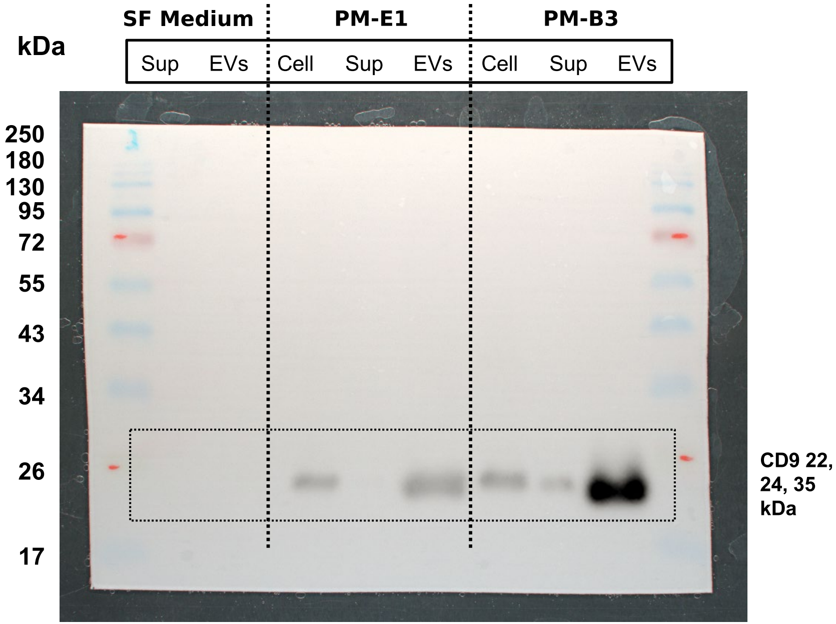

Uncropped blot merged with protein ladder
The same blots were probed with anti-CD9.The signal from Alix emains visible in the top part, exposure time 1 second.

Additional File 4.pdf: Uncropped western blot images of EV markers.

Uncropped blots of figure 1B Blot 1 (left)

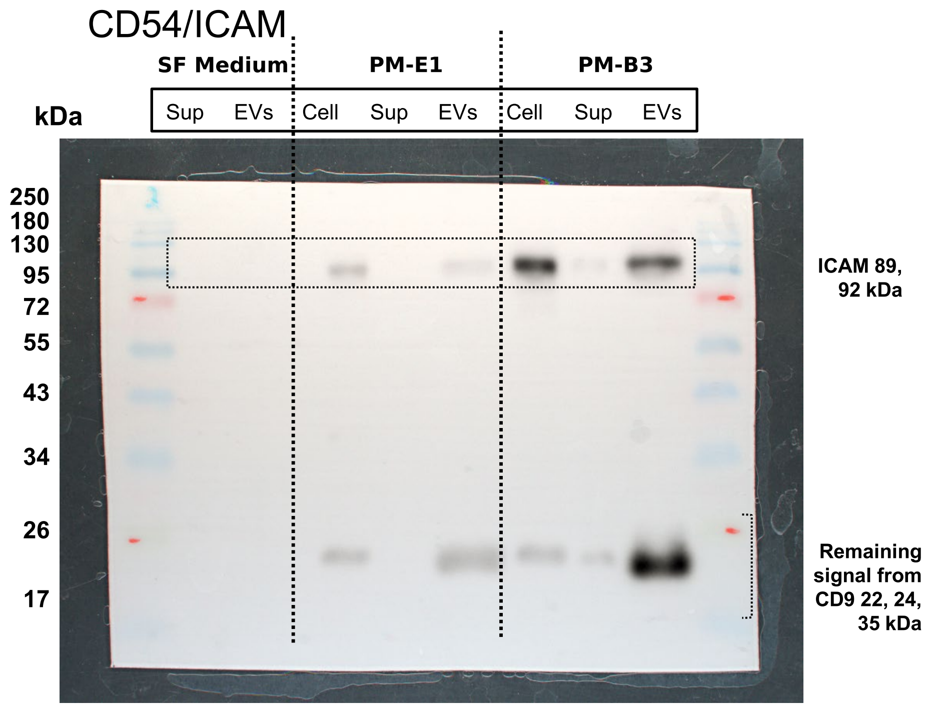

Uncropped blot merged with protein
The blot prevoiusly stained with Alix and CD9 was probed with anti-ICAM antibody, exposure time 1 second. CD9 signal remains visible.

Additional File 4.pdf: Uncropped western blot images of EV markers.

Uncropped blots of figure 1B Blot 2 (top right)

Calnexin

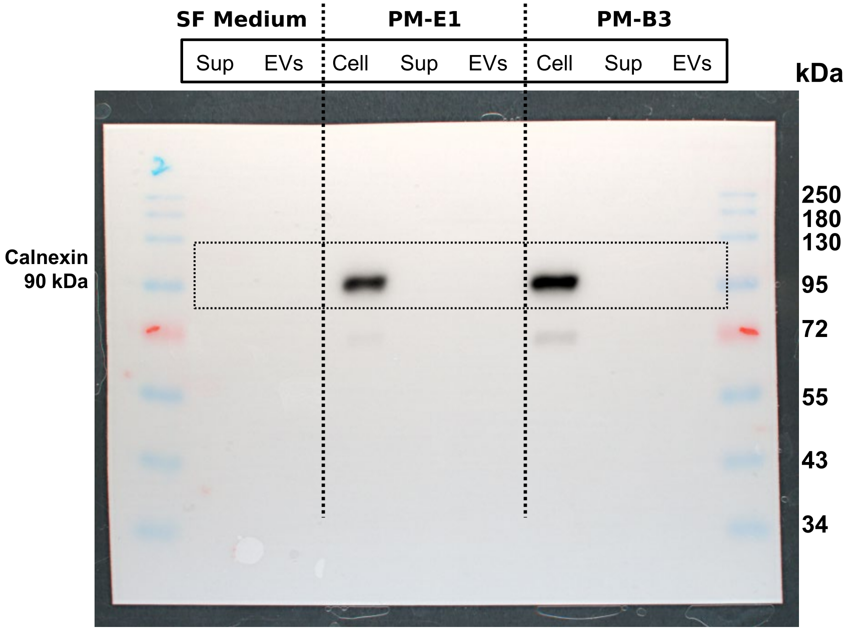

Uncropped blot merged with protein ladder
Fresh blot was cut and stained with anti-Calnexin, exposure time 2 minutes.

Flotillin

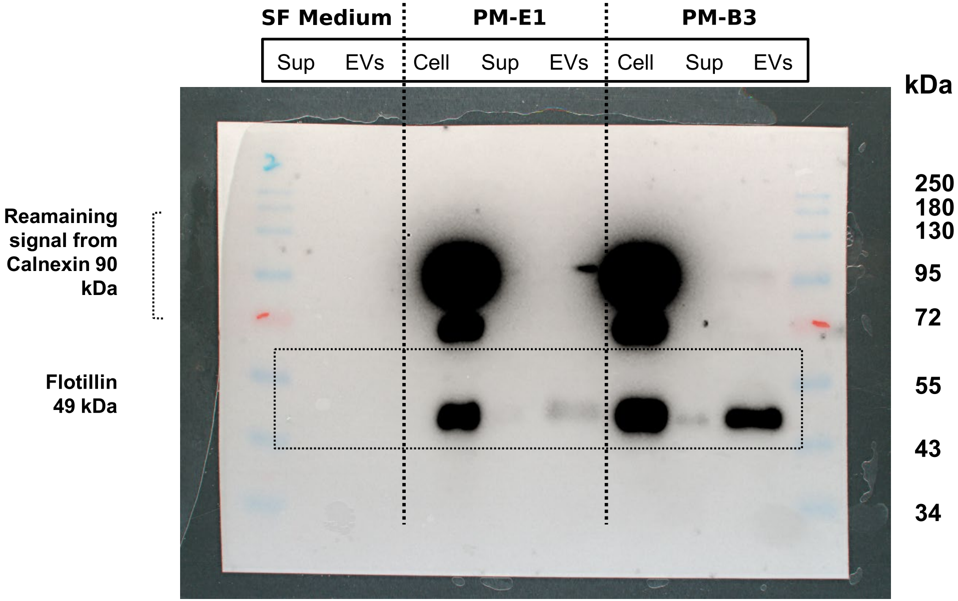

Uncropped blot merged with protein ladder
The same blots were probed with anti-Flotillin antibody, exposure time 2 minutes. Upper bands are the signal from calnexin.

Additional File 4.pdf: Uncropped western blot images of EV markers.

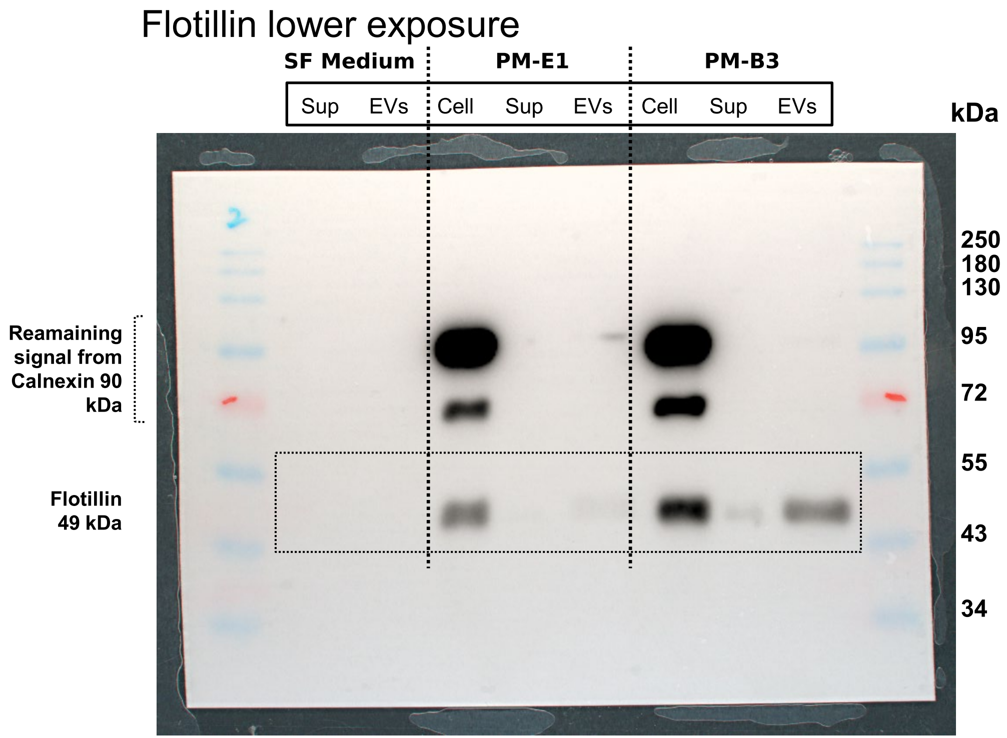

Uncropped blot merged with protein ladder
The same blots were probed with anti-Flotillin antibody, exposure time 1 minute. Upper bands are the signal from calnexin.

Additional File 4.pdf: Uncropped western blot images of EV markers.

Uncropped blots of figure 1B Blot1 (Top)

HSP70

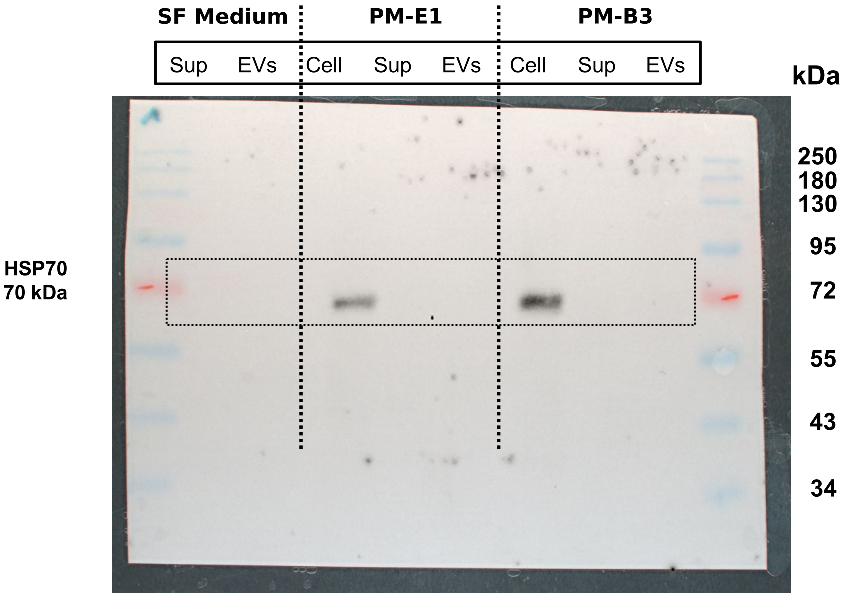

Uncropped blot merged with protein
Fresh blot was stained with anti-HSP70 antibody, exposure time 5 minutes.

GM130

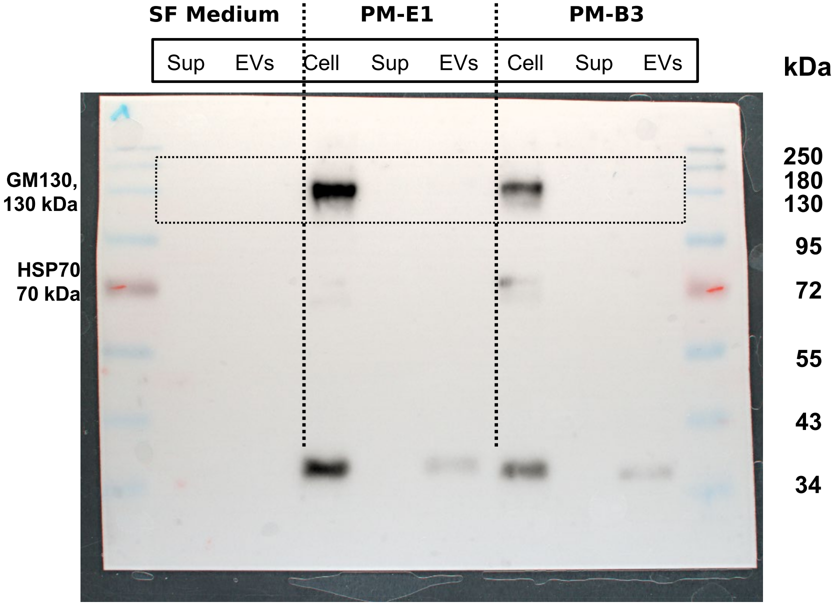

Uncropped blot merged with protein ladder
The same blots were stained with anti-GM130, exposure time 20 seconds. The signal from HSP-70 remains visible.
