# Supplementary material for: Exploring RNA cargo in extracellular vesicles for pleural mesothelioma detection
Source: BMC Cancer. 2025 Feb 7;25:212. doi: 10.1186/s12885-025-13617-y (PMC11804012; doi:10.1186/s12885-025-13617-y)

Additional File 5.pdf: Copy Number Analysis of PM and non-PM cells.

PM cells **PM-E1**

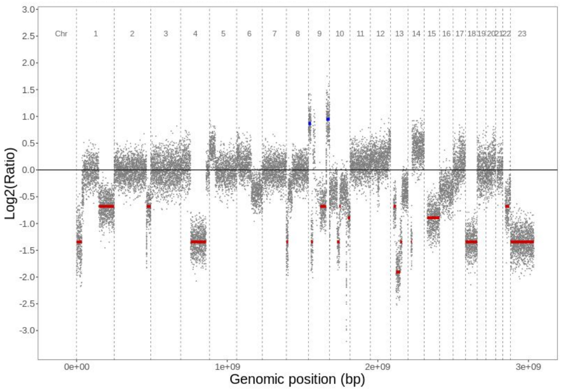

PM tissue **PM-E1**

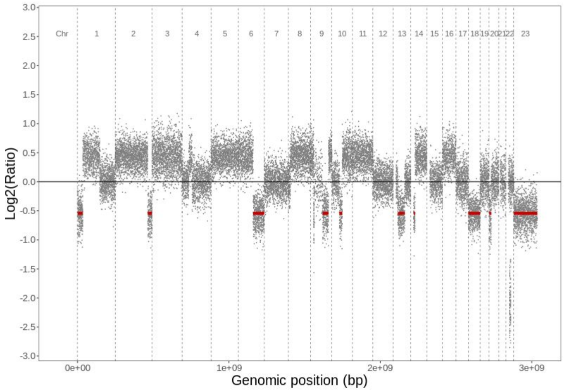

PM cells **PM-B1**

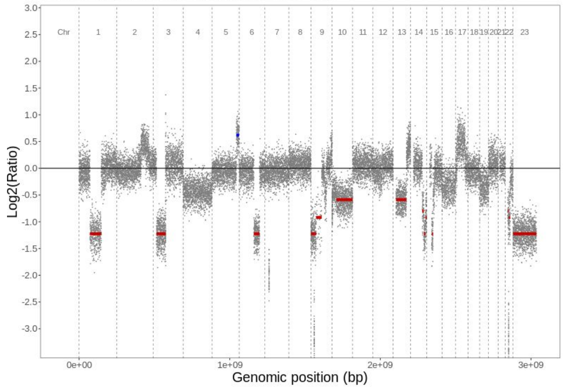

PM tissue **PM-B1**

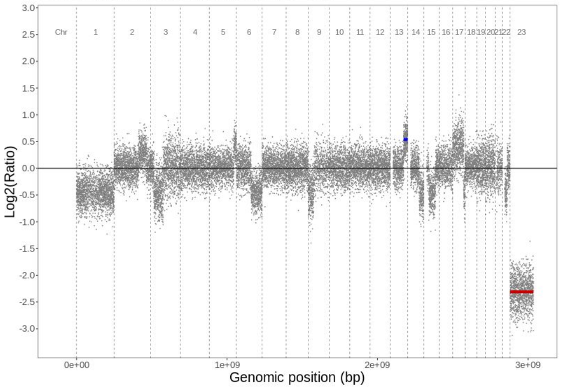

Additional File 5.pdf: Copy Number Analysis of PM and non-PM cells.

PM cells **PM-B2**

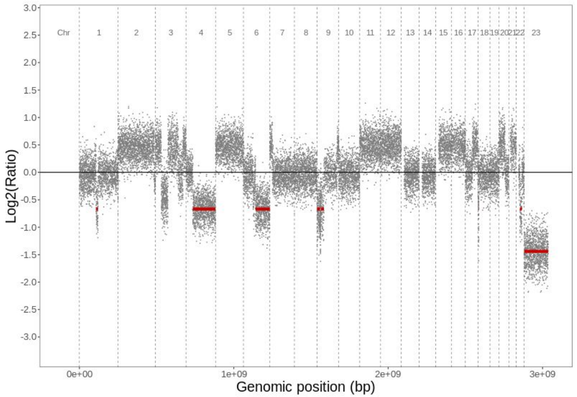

PM tissue **PM-B2**

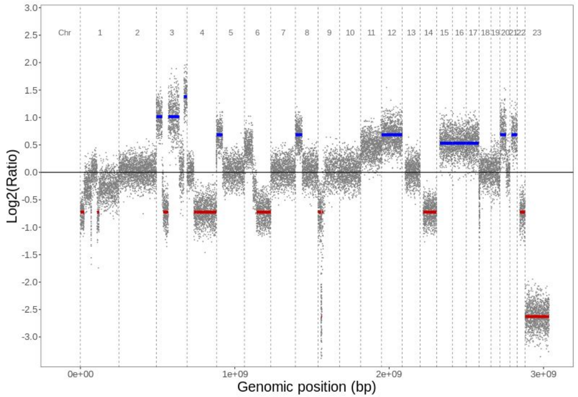

PM cells **PM-B3**

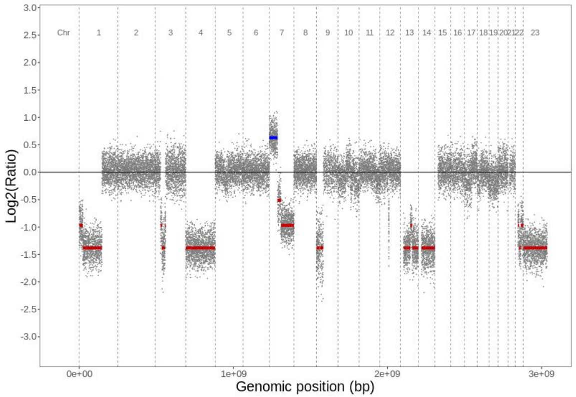

PM tissue **PM-B3**

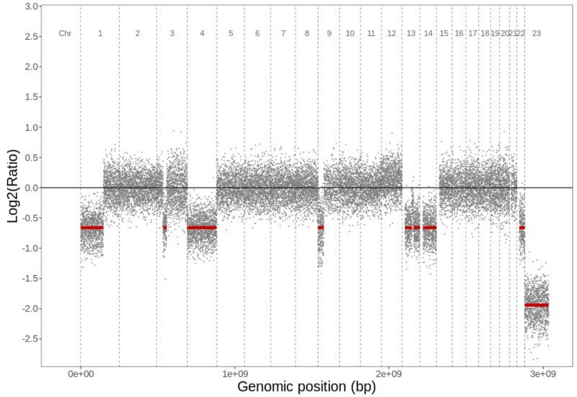

Additional File 5.pdf: Copy Number Analysis of PM and non-PM cells.

non-PM cells **non-PM-1**

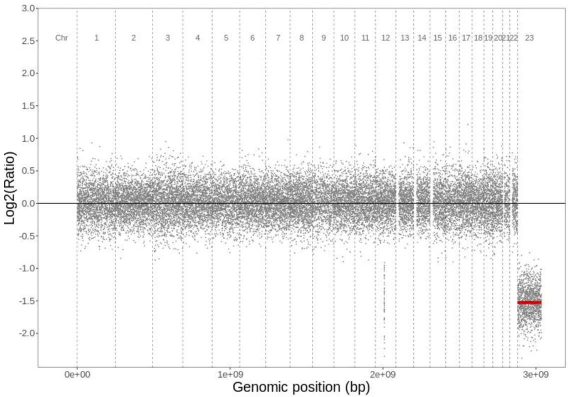

non-PM cells **non-PM-2**

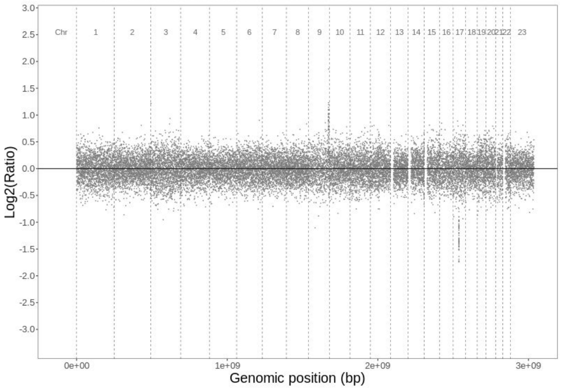

non-PM cells **non-PM-3**

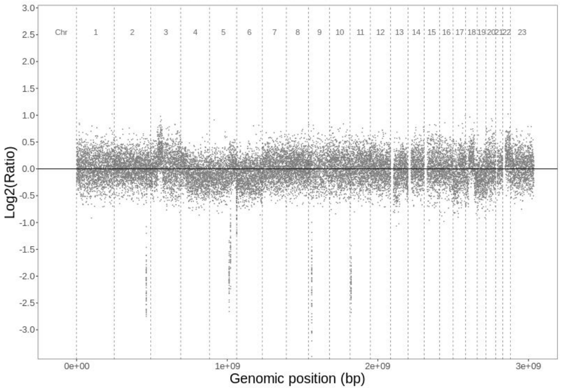

Supplement: Supplementary file 5 — Additional File 5: Copy Number Analysis of PM and non-PM cells. [file 12885_2025_13617_MOESM5_ESM.pdf]
