# Supplementary material for: Exploring RNA cargo in extracellular vesicles for pleural mesothelioma detection
Source: BMC Cancer. 2025 Feb 7;25:212. doi: 10.1186/s12885-025-13617-y (PMC11804012; doi:10.1186/s12885-025-13617-y)

Figure S1: Electrophoresis analysis of RNA extracted from pleural mesothelioma extracellular vesicles.

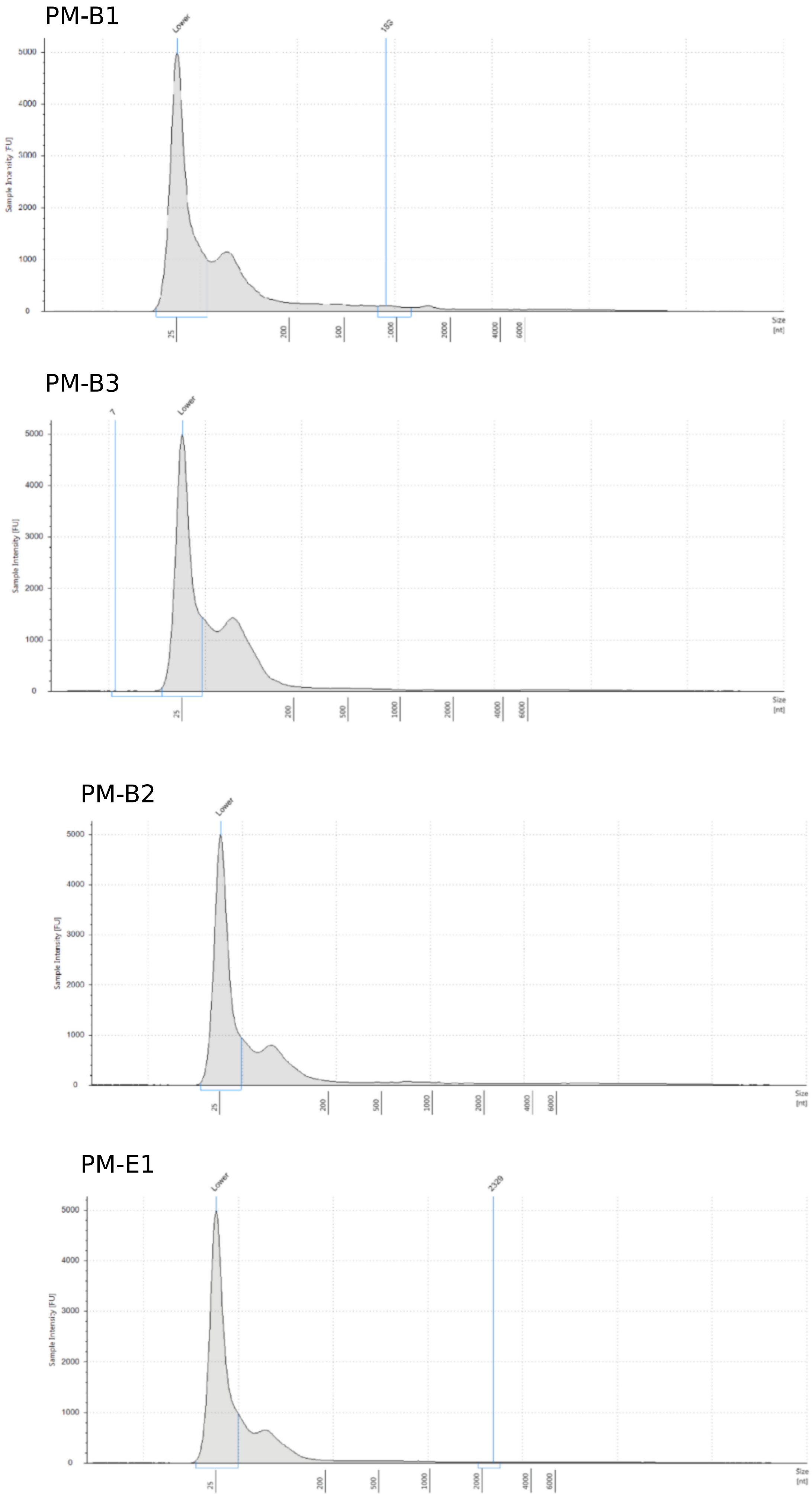

Supplement: Supplementary file 7 — Additional file 7: Figure S1. Electrophoresis analysis of RNA extracted from pleural mesothelioma extracellular vesicles. [file 12885_2025_13617_MOESM7_ESM.pdf]
