# Supplementary figures and images for: Exploring RNA cargo in extracellular vesicles for pleural mesothelioma detection
Source: BMC Cancer. 2025 Feb 7;25:212. doi: 10.1186/s12885-025-13617-y (PMC11804012; doi:10.1186/s12885-025-13617-y)

**Figure S2:** Immunohistochemistry staining of PM and non-PM cells.

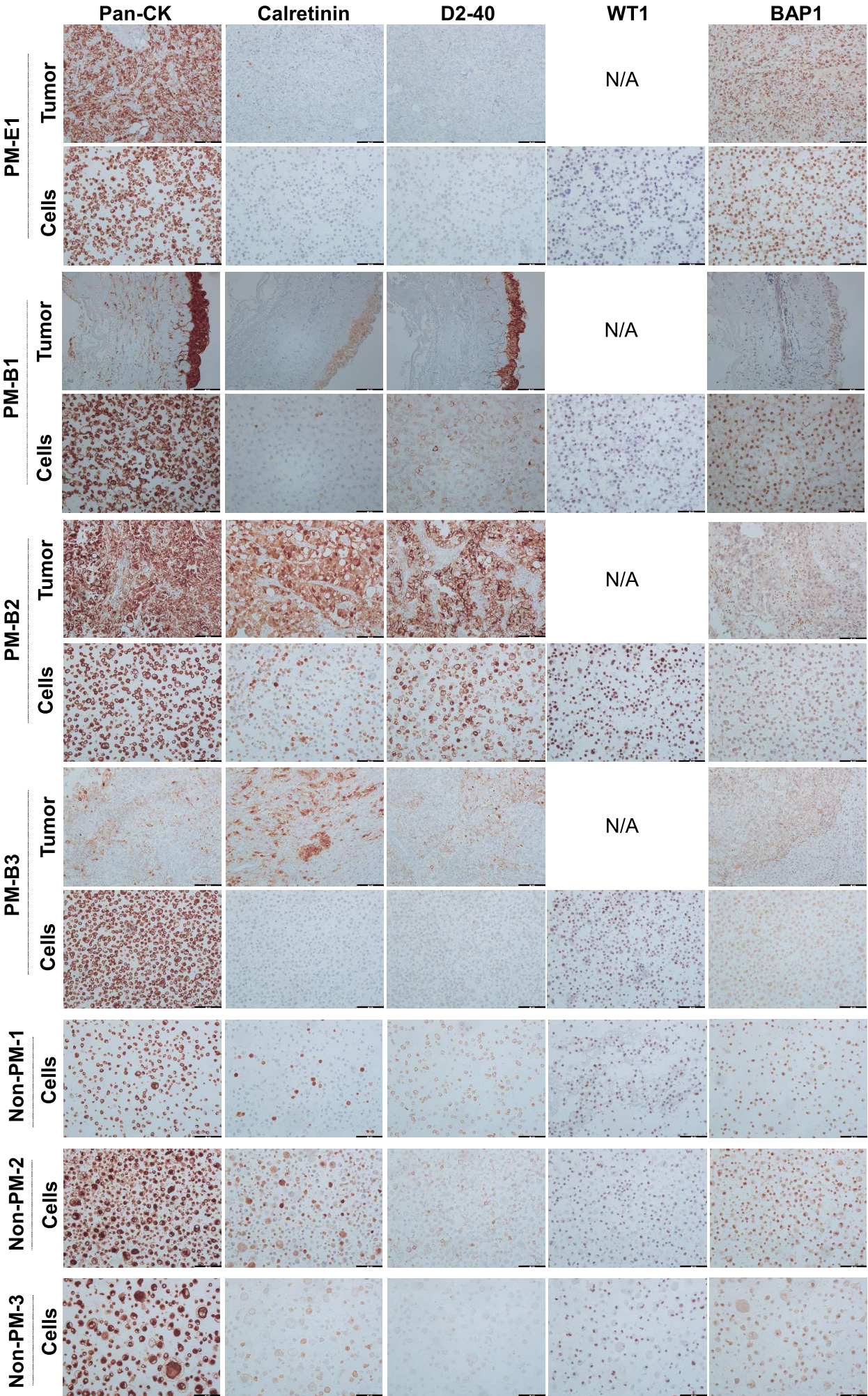

Supplement: Supplementary file 8 — Additional file 8: Figure S2. Immunohistochemistry staining of PM and non-PM cells. [file 12885_2025_13617_MOESM8_ESM.pdf]
